# P2Y_6_ contributes to ovalbumin-induced allergic asthma by enhancing mast cell function in mice

**DOI:** 10.18632/oncotarget.11758

**Published:** 2016-08-31

**Authors:** Jue-ping Shi, Shao-ying Wang, Li-li Chen, Xiao-yu Zhang, Yi-han Zhao, Bing Du, Wen-zheng Jiang, Min Qian, Hua Ren

**Affiliations:** ^1^ Shanghai Key Laboratory of Regulatory Biology, Institute of Biomedical Sciences and School of Life Sciences, East China Normal University, Shanghai, P.R.China

**Keywords:** P2Y_6_, asthma, mast cell, UDP, migration, Immunology and Microbiology Section, Immune response, Immunity

## Abstract

Extracelluar nucleotides have been identified as regulatory factors in asthmatic pathogenesis by activating purinergic receptors. This research aimed to investigate the function of the purinergic receptor P2Y_6_ in mediating airway inflammation in allergic asthma. Wild-type (WT) and *P2Y_6_*-deficient mice were stimulated with ovalbumin (OVA) to construct asthmatic mouse models. Overexpression of P2Y_6_ and uridine 5′-diphosphate (UDP)-releasing were demonstrated in lung tissues in ovalbumin-induced asthmatic mice. The release of the cytokine IL-4, mast cell invasion, and the airway remodeling phenotypes were more severe following the application of UDP in asthmatic mice. However, *P2Y_6_* deficiency reduced these asthmatic pathogeneticsymptoms markedly in a mouse model. *In vitro*, we found that P2Y_6_ in purified mast cells enhanced the functions of mast cells in the inflammatory response in the asthmatic process by triggering their capability for migration, cytokine secretion and granule release. Moreover, P2Y_6_ stimulated the function of mast cells through activation of the AKT signaling pathway. Our data provides evidence that P2Y_6_ contributes to allergic airway inflammation and remodeling by enhancing the functions of mast cells in ovalbumin-induced asthmatic mice.

## INTRODUCTION

Asthma is a kind of chronic lung disease characterized by airway inflammation, remodeling, and hyperresponsiveness [1–3]. In most cases, asthma is derived from sensitization via the airways to some common sensibiligen [4, 5]. They will cause airway inflammation through the release of inflammatory mediators, which is assumed to be the initiating event of airway remodeling. In allergic airway inflammation, including the innate and adaptive immune responses, eosinophils, macrophages, T helper type 2 (Th2) cells, and mast cells are involved in the inflammatory response of asthma [3, 6, 7]. Mast cells play key roles in the allergic inflammation response, airway remodeling and symptomatology [8]. Hence, the mechanism of function of mast cells in asthma is being actively studied now.

In the process of asthma, different inflammatory mediators, including cytokines, chemokines, and lipid mediators, will induce an inflammatory response and pulmonary structural changes [2, 9, 10]. In recent years, extracellular nucleotides (UDP, UTP, ATP, and ADP) have been considered as immunomodulatory mediators that have various functions in inflammatory diseases [11, 12]. Extracellular nucleotides exert their actions by activating ionotropic P2X receptors (P2X_1-7_) or transmembrane receptors of the P2 purinergic receptors family (P2Y_1-14_). Recently, P2Y_2_ and P2X_7_ have been found be involved in allergic airway inflammation [13, 14]. Purinergic receptor P2Y_6_, a G protein-coupled receptor activated by UDP or UTP, has also beeen shown to play an important role in inflammatory responses in some diseases [15–17]. P2Y_6_ was found to be overexpressed in some immune cells, including macrophages, neutrophils, and T cells during the inflammatory response [17–19]. Then, this receptor contributes to the mediation of proinflammatory effects, such as immune cell migration and release of cytokines and chemokines. It is suggested that P2Y_6_ also cause the pathogenesis of airway inflammation by activating some immune cells [16, 20]. However, the functions of P2Y_6_ in mediating inflammation were mostly focused on intestinal inflammation, bacterial infection, or vascular inflammation [15, 19, 21]. P2Y_6_ has been reported to have a potential role in pulmonary airway inflammation and remodeling in the development of asthma [16], but the regulatory mechanism of P2Y_6_ in the proinflammatory response in allergic asthma has not been reported and should be investigated in detail.

In the present study, we developed ovalbumin (OVA)-induced asthmatic mouse models and examined the alteration of the inflammatory response and airway morphologic alterations after *P2Y_6_* deficiency. Cytokine release, immune cell invasion, and airway remodeling were measured in *P2Y_6_*-deficient mice following ovalbumin challenge and UDP treatment to clarify the potential function of P2Y_6_ in allergic asthma in mice. In our results, the release of UDP and overexpression of P2Y_6_ were demonstrated in the process of asthma in mice. Then, extracellular UDP induced more mast cell invasion into lung tissues and activated the functions of mast cells to deteriorate airway inflammation and remodeling in asthma by activating P2Y_6_ on mast cells. It indicated that P2Y_6_ enhanced functions of mast cells through the AKT signaling pathway in allergic asthma and deficiency of P2Y_6_ would limit the development of asthma in mice.

## RESULTS

### P2Y_6_ expression and UDP release in ovalbumin-induced asthmatic mice

**Figure 1:**
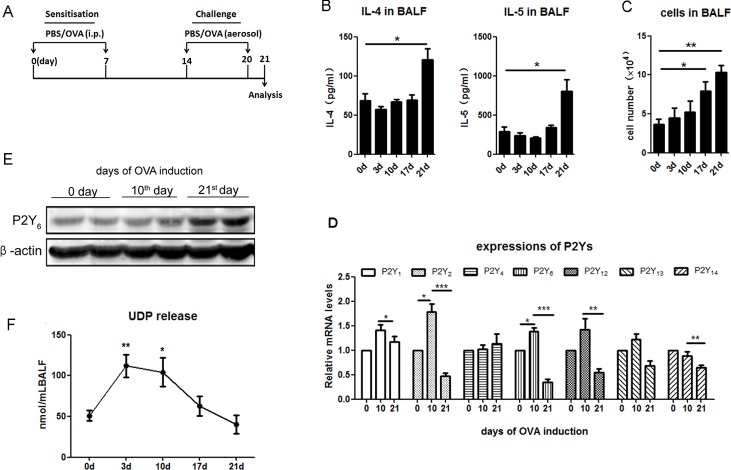
The expressions of P2Ys and UDP release in ovalbumin-induced asthmatic mice **A.** The schematic treatment protocol of ovalbumin treatment for building asthmatic mouse model. **B.** Analysis of released levels of IL-4 and IL-5 in the BALF of ovalbumin-induced asthmatic mice using ELISA method (*n* = 5). **C.** Quantification of total cell invasion in the BALF of ovalbumin-induced asthmatic mice (*n* = 5). **D.** Real-time PCR was used to detect the expression of P2Ys at the mRNA level in ovalbumin-induced asthmatic mice. The relative mRNA levels of different P2Ys receptors were calculated as the method described in “Real-time PCR” of “Materials and Methods”. (*n* = 6) **E.** Detection of P2Y_6_ expression at the protein level in ovalbumin-induced asthmatic mice by western blot. **F.** UDP release in the ovalbumin-induced asthmatic mouse is checked by fluorescence polarization (*n* = 4). * *P* < 0.05, ** 0.01 < *P* < 0.05, *** *P* < 0.01. UDP is the abbreviation of uridine 5′-diphosphate; OVA is the abbreviation of ovalbumin.

To ensure the function of P2Y_6_ in airway inflammation, we developed ovalbumin-induced allergic asthmatic mice (Figure 1A). Firstly, the expression levels of cytokines and leukocyte invasion were detected to evaluate inflammation in ovalbumin-induced asthmatic mice. The ELISA results showed that the levels of IL-4 and IL-5 in bronchioalveolar lavage fluid (BALF) were increased on the 21^st^ day after stimulation with 100 μg ovalbumin (Figure 1B). Then, the number of leukocytes in BALF was quantified using a blood cell counting plate. The results indicated that the invasion of leukocytes into the lung tissues showed time-dependence with ovalbumin induction in asthmatic mice (Figure 1C). To investigate whether P2Ys play roles in the development of asthma, we used real-time PCR to detect P2Ys expressions in mRNA levels in the lung tissues, and found that the expressions of P2Y_1,2,6,12,13_ were increased on the 10^th^ day during ovalbumin treatment, especially that of P2Y_2_ and P2Y_6_. It was implied that these receptors were involved in airway inflammation. However, the expression of P2Y_4_ did not change markedly during ovalbumin treatment (Figure 1D). The expression of P2Y_6_ at the protein level was further confirmed by western blot. It was shown that P2Y_6_ expression was also increased at the protein level on the 21^st^ day during asthma development in mice, but increased mRNA expression occurred later (Figure 1E). Fluorescence polarization was used *in vivo* to test the release level of UDP as the P2Y_6_ agonist during asthmatic development in mice. The results proved that the release of UDP was accompanied by alteration of expression of P2Y_6_ (Figure 1F). The peak of UDP release was on the 3^th^ day of ovalbumin stimulation and after 10 days, its release level was decreased gently. It turned out that UDP activated the function of P2Y_6_ in the process of asthma in mice.

### P2Y_6_ was involved in immune cell invasion in ovalbumin-induced asthmatic mice

**Figure 2:**
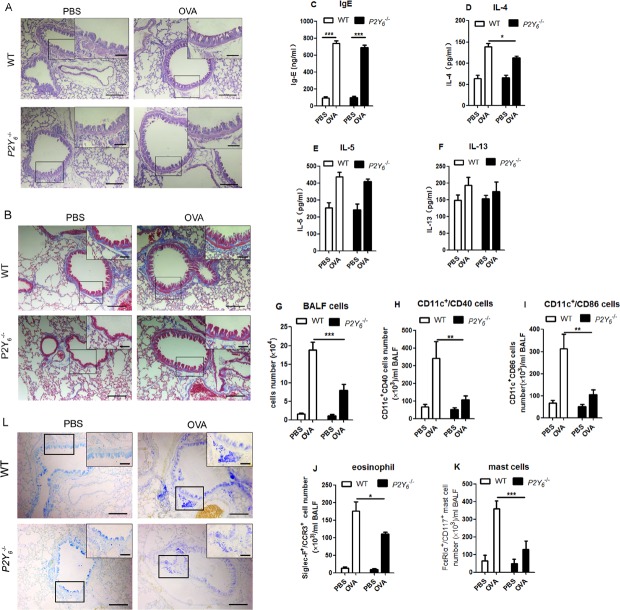
*P2Y*_**6**_ deficiency alleviates ovalbumin-induced inflammation in mice **A.** PAS staining shows goblet cells in ovalbumin and saline-challenged mouse lung tissue. **B.** Masson's trichrome staining is used to determine subepithelial fibrosis in the lung tissues of ovalbumin and saline-challenged mice. **C.**-**F.** ELISA method is used to detect the level of IgE expression in serum and IL-4, IL-5 and IL-13 levels in the BALF in ovalbumin and saline-challenged mice. **G.** The total number of cells in BALF was counted in a hemocytometer using an optical microscope. **H.**-**K.** Flow cytometry is used to quantify the dendritic cell, eosinophil and mast cell numbers in the BALF in ovalbumin and saline-challenged mice. **L.** The situation of mast cell invasion in the lung tissue was analyzed with toluidine blue stains. The lung sections were observed under a light microscopy at 10×magnification and in the upper right corner of each picture is at higher magnification of 40×. Scale bars: 100μm in the panels. * *P* < 0.05, ** 0.01 < *P* < 0.05, *** *P* < 0.01. WT is the abbreviation of wild type; OVA is the abbreviation of ovalbumin.

To study the role of P2Y_6_ in ovalbumin-induced airway conformation and inflammation, we used wild type and *P2Y_6_*^−/−^ littermates to build asthmatic mouse models. Firstly, the goblet cell hyperplasia and subepithelial fibrosis in the lungs were observed under a light microscope with PAS and Masson's trichrome staining in all asthmatic mice. In ovalbumin-induced asthmatic mice, there was a greater increase in hyperplasia of the goblet cells and the deposition of extracellular matrix in the lungs. However, these appearances were all relieved visibly in *P2Y_6_*^−/−^ asthmatic mice (Figure 2A, 2B). P2Y_6_ deficiency relieved the airway conformation and mucus production in the development of asthma in mice.

Then we examined whether P2Y_6_ affected the airway construction through inflammatory reactions. We assessed the levels of IgE in serum and T helper type2 (Th2) relative cytokines IL-4, IL-5 and IL-13 in BALF. Although the level of them were increased in ovalbumin-treated mice, there were no striking difference between the wild type and *P2Y_6_*^−/−^ asthmatic mice except IL-4. The level of IL-4 was decreased weakly after *P2Y_6_* knockout in mice (Figure 2C, 2D, 2E, 2F). It indicated that P2Y_6_ influenced cytokine release slightly in the airway inflammatory reactions in asthma.

In association with airway remodeling in asthma are immune cell invasions, which are one of the major sources of released cytokines. Further, we detected the major type of immune cells including dendritic cells (DCs), mast cells and eosinophil invasion in the lungs of asthmatic mice to investigate whether P2Y_6_ has a role in recruiting inflammatory cells in the process of asthma. In ovalbumin-challenged mice, the total number of cells in BALF were much higher than those in the PBS-treated group. Meanwhile, in *P2Y_6_*^−/−^ asthmatic mice, the invasion of cells in the lungs were markedly alleviated compared with wild type asthmatic mice (Figure 2G). Moreover, we analyzed the major types of immune cells in the lungs using flow cytometery. It was found that CD11c^+^/CD40^+^ DC cells, CD11c^+^/CD86^+^ DC cells, Siglec-F^+^/CCR3^+^ eosinophils, and CD117^+^/FcεRIα^+^ mast cells in the BALF were all increased significantly in ovalbumin-induced wild type asthmatic mice and no invasion of these immune cells occurred in the control group. After *P2Y_6_*^−/−^ deficiency, the invasion of these cells in the lungs was decreased exceedingly in asthmatic mice, especially that of mast cells, which are important inflammatory cells in the development of asthma (Figure 2H, 2I, 2J, 2K). Furthermore, toluidine blue staining was used to evaluate mast cell invasion in the lung tissues and the same phenomenon was observed, in line with our previous result. Ovalbumin induced mast cell invasion in the lungs in wild type asthmatic mice, but this situation was relieved in *P2Y_6_*-deficient asthmatic mice (Figure 2L). The result revealed that P2Y_6_ enhanced inflammatory cell invasion, especially mast cells, in ovalbumin-induced airway inflammation in mice.

### P2Y_6_ was activated by UDP to enhance ovalbumin-induced airway inflammation in mice

**Figure 3:**
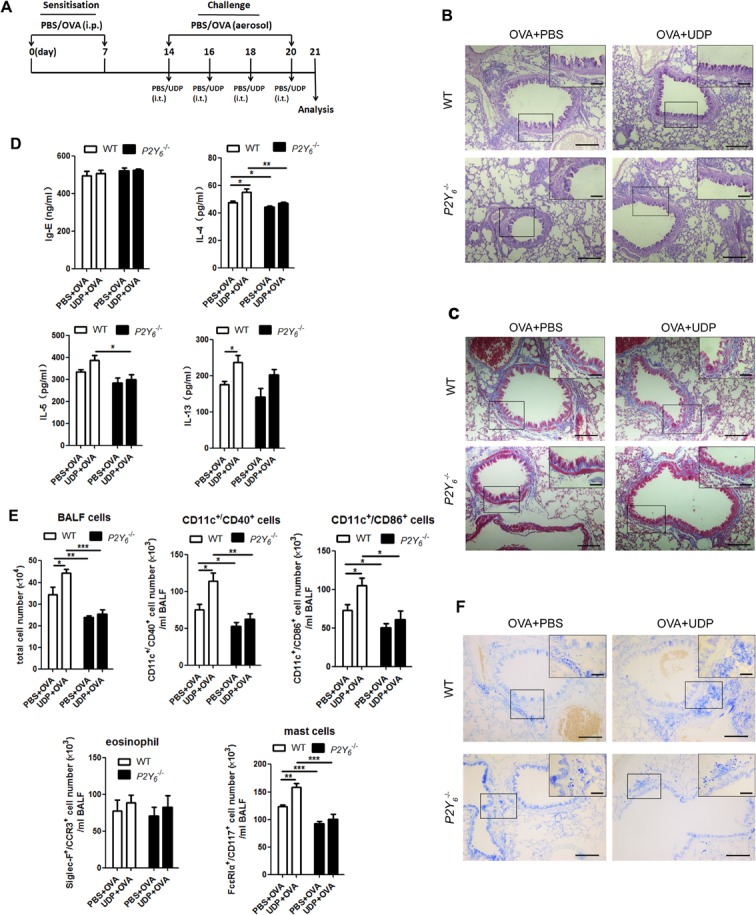
UDP enhance inflammation in ovalbumin-induced asthmatic mice **A.** The schematic protocol of UDP treatment in ovalbumin-induced asthmatic mouse model. PAS staining **B.** and Masson's trichrome staining **C.** results for lung tissues in ovalbumin-challenged mice with or without UDP treatment. **D.** The IgE level in serum and levels of IL-4, IL-5 and IL-13 in the BALF were analyzed using ELISA in ovalbumin-induced asthmatic mice with or without UDP treatment. **E.** The total numbers of cells in the BALF were quantified for ovalbumin and UDP-treated wild type or *P2Y*^−/−^ mice using a blood cell counting plate. Flow cytometry was used to quantify the numbers of dendritic cells, eosinophils, and mast cells in ovalbumin and UDP-treated wild type or *P2Y*^−/−^ mice. **F.** Toluidine blue stains was used to show the mast cell invasion in the lung tissues in mice. Scale bars: 100μm in the panels. * *P* < 0.05, ** 0.01 < *P* < 0.05, *** *P* < 0.01. WT is the abbreviation of wild type; UDP is the abbreviation of uridine 5′-diphosphate; OVA is the abbreviation of ovalbumin.

UDP, a P2Y_6_ agonist *in vivo*, was released during the process of ovalbumin stimulation in mice in our previous experiment. Here we intend to study whether UDP activates its purinergic receptor P2Y_6_ to affect ovalbumin-induced airway remodeling and inflammation in mice. We also used ovalbumin to induce allergic inflammation in littermates wild type and *P2Y_6_*^−/−^ mice and meanwhile added UDP stimulation intratracheally after atomization for 30 min every three days during the process of model construction (Figure 3A). The results showed greater goblet cell hyperplasia in the airway epithelial layer in UDP-treated wild type asthmatic mice than in asthmatic mice, but not in *P2Y_6_*^−/−^ asthmatic mice (Figure 3B). Unfortunately, a similar situation of deposition of extracellular matrix was found in UDP-treated asthmatic mice compared with single ovalbumin-induced mice, regardless of whether *P2Y_6_* were deficiency (Figure 3C).

Then we analyzed the alteration of airway inflammation caused by UDP in asthmatic mice, including the levels of IgE in serum, IL-4, IL-5 and IL-13 in BALF. As shown in Figure 3D, UDP did not affect the altering of IgE level in serum and there is no difference of that between wild type and *P2Y6*^−/−^ mice. UDP enhanced the expression of IL-4, IL-5 and IL-13 in BALF in ovalbumin-treated wild type mice, and after *P2Y_6_* deficiency, it caused reduction of the levels of IL-4 and IL-5 in BALF. As a proof of concept, more immune cells will influence cytokine release and allergic airway inflammation in the lungs. In this regard, the invasion of DCs, mast cells, and eosinophils in the lungs were measured after UDP treatment in asthmatic mice. We found that more immune cells invaded the lungs induced by UDP and ovalbumin together in mice, especially mast cells (Figure 3E). However, no more cells were observed in the lung in ovalbumin-sensitized *P2Y6*^−/−^ mice, even those treated with UDP. In addition, UDP did not impact eosinophil invasion in the lungs of mice during the process of asthma. To verify the role of UDP in mast cell invasion, we further used toluidine blue staining to observe the mast cells in the lungs of asthmatic mice with and without *P2Y_6_* deficiency (Figure 3F). According to the results, more mast cells were observed in the lung tissues of the UDP-treated asthmatic mice group and this appearance was reduced after *P2Y_6_* deficiency. Therefore, P2Y_6_ activated by UDP enhanced mast cell invasion and IL-4 release to modulate mucus hypertrophy in the development of asthma in mice.

### Activation of P2Y_6_ with UDP increased the function of mast cells *in vitro*

**Figure 4:**
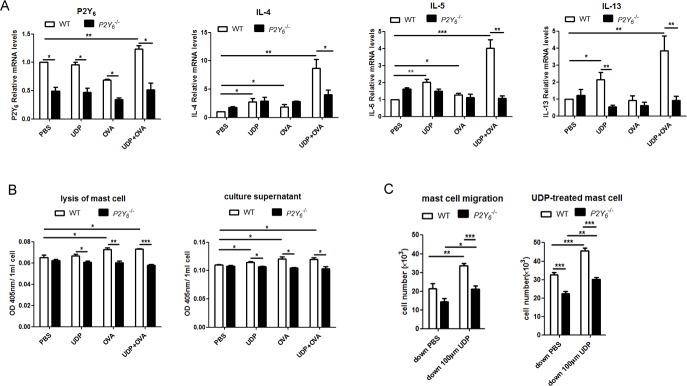
Analysis of the function of *P2Y*_**6**_ activated by UDP on mast cells *in vitro* **A.** Detection of the expression of P2Y_6_, IL-4, IL-5 and IL-13 in mast cells after stimulation with ovalbumin and UDP by real-time PCR method. **B.** Mast cell degranulation ability was measured by assessing hexosaminidase activity in the culture supernatant or cell lysates. **C.** The chemotactic ability of mast cell was determined by the transwell method. * *P* < 0.05, ** 0.01 < *P* < 0.05, *** *P* < 0.01. WT is the abbreviation of wild type; UDP is the abbreviation of uridine 5′-diphosphate; OVA is the abbreviation of ovalbumin.

To investigate the regulatory function of P2Y_6_ in mast cells in asthma, mast cells were induced with IL-3 and SCF from primary separation of bone marrow cells, then stimulated with IgE overnight before treatment with UDP and ovalbumin in *vitro*. Firstly, alteration of the expression of P2Y_6_ at the mRNA level was detected in mast cells after stimulation with ovalbumin and UDP. The results indicated that this purinergic receptor on mast cells was overexpressed when treated with UDP and ovalbumin acting cooperatively (Figure 4A). Additionally, after activated mast cells were treated with UDP and ovalbumin together, the levels of inflammatory cytokines, including IL-4, IL-5, and IL-13, were increased compared with PBS treated, UDP-induced, and ovalbumin-induced groups (Figure 4A). However, in *P2Y_6_*^−/−^ mast cells, no significant alteration of these cytokines was found when the cells were treated with UDP, ovalbumin or the two together. Therefore, we believe that in the process of ovalbumin stimulation, IgE-primed mast cells released more cytokines induced by UDP through the P2Y_6_ receptor on mast cells. Then we detected whether UDP stimulated the degranulation ability of mast cells during ovalbumin stimulation. The activity of β-hexosaminidase released from mast cells was analyzed to determine the function of degranulation in mast cells. Our evidence indicated that UDP also increased granule release from mast cells. Nonetheless, after *P2Y_6_* knockout in mast cells, no enhancement of degranulation ability was observed when cells were induced with UDP or ovalbumin (Figure 4B). In our previous experiments, greater mast cell invasion in lung tissues was found in ovalbumin-induced mice additionally treated with UDP. Here, we detected whether UDP impacts the ability of chemotactic migration of mast cells *in vitro*. When 100 μm UDP was added to the lower compartment of transwell inserts, it induced a significant increase of mast cell migration to the lower chambers (Figure 4C). But this chemotactic response of mast cells was reduced after *P2Y_6_* deficiency. In order to confirm the function of P2Y_6_ on UDP-induced migration of mast cells, the mast cells were treated with UDP for 30 min to activate the P2Y_6_ receptor before chemotactic assays. Then, the activated mast cells were added into the upper chambers of transwell inserts. According to the results of cell counting, an obviously increasing number of mast cells was also induced with UDP in the lower chamber for 3 h. In Figure 4C, it is shown that the migration of mast cells from upper to lower chambers was decreased after *P2Y_6_*^−/−^ knockout, even without UDP induction. Here, our results indicated that P2Y_6_ on mast cells activated by extracellular UDP enhanced the functions of mast cells, including cytokine secretion, degranulation, and migration abilities, which affected the development of asthma.

### UDP regulates the function of mast cells through the AKT signal pathway

**Figure 5:**
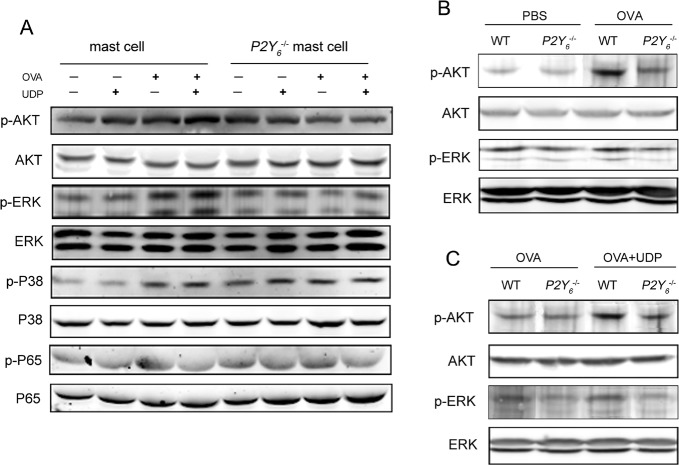
*P2Y*_**6**_-related signaling pathway detection by western blot in mast cells **A.** The phosphorylation levels of P65, AKT, ERK, and P38 in mast cells were determined by western blot. **B.** The phosphorylation levels of AKT and ERK in the lung tissues in ovalbumin-induced wild type and *P2Y*^−/−^ mice. **C.** The phosphorylation levels of AKT and ERK in the lung tissues in UDP-treated wild type and *P2Y*^−/−^ asthmatic mice. WT is the abbreviation of wild type; UDP is the abbreviation of uridine 5′-diphosphate; OVA is the abbreviation of ovalbumin.

To explore the mechanism of UDP-induced enhancement of function of mast cells through its special receptor P2Y_6_, the phosphorylation of AKT, ERK, P38 and P65 was analyzed by western blot assay to assess the UDP/P2Y_6_-related signal pathway in mast cells. As shown in Figure 5A, the phosphorylation of AKT, ERK, and P38 was increased in mast cells induced by UDP, ovalbumin, and the two together. Meanwhile, in *P2Y_6_*^−/−^ mast cells, the increase in phosphorylation of P38 was also induced by UDP and ovalbumin. The UDP and ovalbumin induced increased phosphorylation of AKT and ERK was decreased after *P2Y_6_*^−/−^ deficiency in mast cells. These results suggested that AKT and ERK might be involved in the UDP/P2Y_6_-mediated functions of mast cells in ovalbumin-induced asthma *in vitro*. In order to confirm the regulatory pathway of UDP/P2Y_6_ in its entirety, the levels of phosphorylated proteins were detected in the lung tissues of ovalbumin-induced asthmatic mice (Figure 5B). A sharp increase in the phosphorylation of AKT was found in ovalbumin-induced mice and this phenomenon was reduced observably in *P2Y_6_*^−/−^ deficient mice. Moreover, the same alteration in phosphorylation of AKT occurred in the lung tissues of UDP-treated ovalbumin-induced asthmatic mice (Figure 5C). Thus, our results indicated that the AKT-related signaling pathway was more critical for regulatory function of UDP/P2Y_6_ on mast cells in the development of asthma. The molecular mechanism of how AKT signaling activates the function of mast cells still needs to be further explored.

## DISCUSSION

As it is known, inflammatory mediators, such as Th2 cells, producing IL-4, IL-5 and IL-13 contribute to not only inflammation but also airway remodeling in asthma [22, 23]. IgE is considered a triggering antibody for the allergic response through activation of mast cells and basophils [24]. Recent years, extracellular nucleotides (UDP, UTP, ATP, and ADP) known as danger signals have been found to play important roles in inflammatory response as immunoregulatory mediators, including in pulmonary inflection [11, 20]. Some of these nucleotides take out their functions by activating P2Y purinergic receptors, which are expressed on different immune cells. In asthmatic airway inflammation, ATP was reported to mediate the P2Y_2_ receptor in triggering dendritic cell and eosinophil recruitment and reactive oxygen species production [14, 25]. However, the functions of P2Y receptors involved in asthma to other extracellular nucleotides remains insufficiently understood. In our ovalbumin-induced asthmatic mice, the expression of not only P2Y_2_ but also P2Y_6_ changed sharply during the development of asthma. The expression of P2Y_6_ was increased in the ovalbumin-challenged stage and was shown to be decreased in the later stimulated phase. Meanwhile, the same variable tendency to release UDP in lung tissues was found in the asthmatic mice (Figure 1). According to our results, UDP release induced more activation of its specific purinergic receptor P2Y_6_, which implied that P2Y_6_ was involved in asthmatic airway inflammatory development in mice.

The presence of P2Y_6_ transcripts was detected in some immune cells, including macrophages, neutrophils, dendritic cells, T cells, and mast cells, and it was verified to be involved in cytokine secretion and cell migration in proinflammatory reactions [17, 26]. Here, we found over-expression of P2Y_6_ in lung tissues in mice after challenge with ovalbumin, which suggested a possible role in allergic airway inflammation. In order to investigate the role of P2Y_6_ in asthma, *P2Y_6_* -deficient mice were used to construct ovalbumin-induced asthmatic mice. The deficiency of *P2Y_6_* would relieve the phenotype of airway remodeling, such as airway epithelial extensions, goblet cell formation, and subepithelial fibrosis in lung tissues in asthmatic mice. However, P2Y_6_ did not strongly affect the alteration of IgE in serum nor cytokine release, except that of IL-4 in BALF in mice. Further, the total number of immune cells in BALF was decreased markedly after *P2Y_6_* knockout, and among them was mast cells (Figure 2). With the intrapulmonary application of the P2Y_6_ agonist UDP, more severe airway remodeling and inflammation, including immune cell invasion and high levels of cytokines occurred in lung tissues in ovalbumin-induced asthmatic mice (Figure 3). These symptoms were abrogated after P2Y_6_ deficiency. It was demonstrated that P2Y_6_ enhanced immune cell invasion in lung tissues by application of UDP. Here, mast cells invasion in lungs was shown to be reduced mostly in *P2Y_6_*^−/−^ mice. Therefore, we focused on the function of P2Y_6_ in mast cells in our present research. As dendritic cells and eosinophils are also required in the airway inflammatory process [27, 28], the regulatory mechanism of P2Y_6_ in these should be further investigated.

Further, we examined function of P2Y_6_ on mast cells *in vitro* that purified and induced from bone marrow cells in wild type and *P2Y_6_*^−/−^ mice. During a pulmonary allergic response, mast cell progenitors migrate from the blood into lung tissues. IgE released from B cells will activate mast cells to release immune mediators, such as histamine and cytokines, which will cause more serious airway remodeling [8, 29–31]. We found much higher levels of cytokine (IL-4, IL-5, and IL-13) mRNAs in mast cells with stimulation of UDP and ovalbumin together. In addition, the stronger degranulation and migration abilities of mast cells were shown when cells were treated with ovalbumin and UDP acting cooperatively. These proinflammatory capabilities of mast cells were blocked after P2Y_6_ deficiency. Taken together, these results indicate that P2Y_6_ enhanced the functions of mast cells in pro-inflammation reactions in the asthmatic process by triggering migration and cytokine and granule release. These mediators released from mast cells have been shown to cause tissue remodeling, especially in the airway [8, 32].

AKT and ERK signals were involved in degranulation and cytokine secretion in mast cells in allergic asthma [33–35]. Not surprisingly, the phosphorylation levels of AKT and ERK were increased after treatment with ovalbumin for IgE-stimulated mast cells, especially with application of UDP. The increased phosphorylation of AKT and ERK was reduced in *P2Y_6_*^−/−^ mast cells. Next, in lung tissues of ovalbumin-induced asthmatic mice, only an increase in the phosphorylation level of AKT was indicated, including in those treated with UDP, and this increase was inhibited after P2Y_6_ knockout. Thus, P2Y_6_ appeared to be more important in mediating the AKT signaling pathway in asthmatic development in mice. Further study will be undertaken to reveal the regulatory mechanism of P2Y_6_ in AKT signaling in asthma in detail.

In conclusion, P2Y_6_ was evidenced to be involved in allergic airway inflammation and remodeling in asthmatic mice by enhancing the function of mast cells, such as migration into lung tissues, degranulation ability and cytokine secretion. Interestingly, in mast cells and lung tissue, P2Y_6_ deficiency will reduce IL-4 expression most obviously in ovalbumin-induced asthma. In asthma, IL-4 released from Th-2 cells contributes to stimulate proliferation and differentiation of B cells that release IgE to mast cells [27, 36]. Mast cells also secrete IL-4 to cause allergic inflammation and modulate pulmonary endothelial cell alteration [8]. According to our results, P2Y_6_ increased IL-4 release during the process of asthma and the function of P2Y_6_ on IL-4 should be actively studied. Further, UDP, the agonist of P2Y_6_, was found to aggravate asthmatic symptoms as an inflammatory mediator in lung tissues in mice by mediating the function of mast cells. Therefore, more research needs to investigate further to reveal the mechanism of P2Y_6_ regulating mast cells in the pathogenesis of asthma for finding innovate treatments of this disease.

## MATERIALS AND METHODS

### Animals

Wild-type C57BL/6 mice were purchased from the Shanghai Laboratory Animal Company (Shanghai, China) and *P2Y_6_*^−/−^ C57BL/6 mice were obtained from the Laboratory Animal Center of East China Normal University (Shanghai, China). All mice were bred and housed under pathogen-free conditions and maintained according to institutional guidelines. The mice were 6-8 weeks old at the time of the experiments and they were sacrificed using the method of cervical dislocation before experiments. All experimental protocols were approved by the Animal Investigation Committee of East China Normal University. Age-matched littermates (6-8 weeks old) with different genotypes were used for ovalbumin-induced asthmatic mouse models. Before the experiments, *P2Y_6_* deficiency in the mice was confirmed by PCR.

### Ovalbumin sensitization and challenge in mice

For the construction of a suitable asthma mouse model, a sensitization concentration of 100 μg ovalbumin (grade V, Sigma-Aldrich, St. Louis, USA) plusing 1% Al(OH)_3_ in a volume of 200 μL sterile PBS was intraperitoneally injected into different groups of wild type and *P2Y_6_*-deficient mice on day 0 and 7(Figure 1A) [37]. On days 14 to 20, sensitive mice were challenged by atomization with 1% ovalbumin in PBS or PBS only as control for 30 min each time. The UDP-stimulated group were injected intratracheally (i.t.) with 1 mM UDP (Sigma-Aldrich, St. Louis, USA) or PBS as a control at 30 min after atomization on days 14, 16, 18, and 20 (Figure 3A). At 24 h after the last challenge, serum, bronchoalveolar lavage fluid and lung tissues were collected after all mice were euthanized.

### Bronchoalveolar lavage fluid leukocyte count

Lungs were flushed twice with cold 0.5% fetal bovine serum in 1 mL PBS. BALF was obtained after lavage and centrifuged at 2000 g at 4°C for 5 min. The depositions were resuspended in 50 μL PBS, and the total number of cells was counted in a hemocytometer.

### IgE and cytokine analysis

The serum samples were obtain by centrifugation of blood samples at 3000 g at 4°C for 10 min. Then the levels of total IgE in serum were analyzed with a commercial enzyme-linked immunosorbent assay (ELISA) kit (Biolegend, California, USA). The levels of interleukin IL-4, IL-5 and IL-13 in the BALF were detected using the corresponding ELISA kit (Biolegend, California, USA) following manufacturer's instructions.

### Histology and immunohistochemistry

To analyze goblet cell hyperplasia, the left upper lung from each mouse was fixed in 4% paraformaldehyde overnight (24 h) in dimethylbenzene and then rehydrate them in decreasing concentrations of ethanol. The slices were incubated in periodic acid alcohol before stained with periodic acid-Schiff (PAS) reagent [38]. Then, they were washed with sulfurous acid, followed by nucleolus staining using hematin and mounting in glycerol. PAS-positive cells and mucus secretion in the lungs were observed under a light microscope.

The lung tissue slices were incubated in periodic acid alcohol before observing subepithelial fibrosis in the lungs. The slices were washed with deionized water, and nucleolus were stained using hematoxylin. Then the subepithelial fibrosis in lung tissues were determined by Masson's trichrome-stained reagent staining (Zhongshanjinqiao, Beijing, China) [39]. The slices were observed under a light microscope after mounting in glycerol.

To observing the mast cells in the lung tissue, the lung tissue slices were dewaxed in dimethylbenzene and rehydrated in decreasing concentrations of ethanol. Then stained with toluidine blue and wash with glacial acetic acid [40]. The slices were observed under a light microscope after mounting in glycerol.

### Ovalbumin sensitization and cell challenge

After the primary separation of bone marrow cells from femora and tibiae of wild-type mice, 10 ng/ml IL-3 and 10 ng/mL SCF (Biolegend, California, USA) were added into RPMI-1640 cell culture medium to induce bone marrow cell differentiation to mast cells. After 4-6 weeks culture, flow cytometry analysis was used to separate CD117 and FcεRIα-positive mast cells (APC anti-CD117 and FITC anti-FcεRIα, Biolegend, California, USA) [41]. The purified mast cells were activated using human IgE full-length protein (Abcam, Massachusetts, USA) overnight and then challenged with 200 μg ovalbumin for 1 h [42]. Real-time PCR was used to detect the mRNA expression levels of P2Y_6_, IL-4, IL-5 and IL-13 on mast cells.

### Flow cytometry analysis and cell sorting

The BALF were incubated with different antibodies for 1 h: FITC anti-CCR3, PE anti-Siglec-F for eosinophil [43], FITC anti-FcεRIα, and APC anti-CD117 for mast cells, FITC anti-CD11c, and APC anti-CD40 and APC anti-CD86 for DCs (BD Biosciences, New Jersey, USA or Biolegend, California, USA). After the BALF were washed by PBS three times, the different types of cells were analyzed and sorted using a flow cytometer (BD FACSAria, USA) and data were analyzed using CellQuest software (BD Biosciences, USA).

### Western blot

After treatment or stimulation, the lung tissues were lysed by RIPA buffer (Cell Signaling Technology, USA). The concentration of total protein was measured by BCA assay (Pierce) and adjusted to the same concentration with extraction reagent. Samples were heated at 100°C for 5 min and loaded onto 10% SDS-PAGE. After electrophoresis, the gel was transferred onto polyvinylidene fluoride (PVDF) membrane and blocked with 5% BSA. The membrane was incubated with primary Abs and then with the appropriate fluorescent secondary Abs. Subsequently, the immunoreactive proteins were detected using the Odyssey laser digital imaging system (Gene Company, Hongkong, China). The primary antibodies including anti-AKT, anti-phospho-AKT, anti-ERK, anti-phospho-ERK, anti-P38, anti-phospho-P38, anti-P65 and anti-phospho-P65 are all rabbit monoclonal antibodies (Cell Signaling Technology, USA). The P2Y6 antibody is a rabbit polyclonal IgG (Santa Cruz Biotechnology, USA). The secondary Antibody is IRDye^®^ 680LT Goat anti-Rabbit IgG (LI-COR Bioscience, USA).

### Real-time PCR

The sorted cells were stored in the TRIzol (TaKaRa Clontech, Japan). Total RNAs were extracted according to the manufacturer's protocol of TakaRa. Template cDNAs were obtained by reverse transcription of total RNA using PrimeScript™ RT reagent Kit with gDNA Eraser (TaKaRa Clontech, Japan). Amplification was carried out by using SYBR^®^ Premix Ex Taq™ (Tli RNaseH Plus) (TaKaRa Clontech, Japan). The mRNA expression level of β-actin was used as internal control. The relative mRNA levels of P2Y_s_ were calculated as follows [44]. P2Ys genes were taken for example and the average CT for β-actin was subtracted from the average value for P2Ys to generate Δ for each sample. CT is the cycle number of PCR amplification. Δ = CT× (P2Ys) - CT× (β-actin). Therefore, ΔΔ = Δ(P2Ys value for the samples at day10 or day21)- Δ(P2Ys value for the samples at day0). Finally, the formula 2^−ΔΔ^ was taken to calculate the relative mRNA level compared with the control. The sequences of the primers for real-time PCR are listed in Table 1.

**Table 1:** Sequences of the primers for real-time PCR

Gene name	Primers (5′- 3′)
Il-4	forward: GGTCTCAACCCCCAGCTAGT
	reverse: GCCGATGATCTCTCTCAAGTGAT
Il-5	forward: AGGCTTCCTGTCCCTACTCA
	reverse: AATCCAGGAACTGCCTCGTC
Il-13	forward: CCTGGCTCTTGCTTGCCTT
	reverse: GGTCTTGTGTGATGTTGCTCA
P2Y_1_	forward: CGCACACAGGTACAGTGGCGT
	reverse: TTCCGAGTCCCAGTGCCAGAGT
P2Y_2_	forward: GTGCGGGGAACCCGGATCAC
	reverse: AGCCGCCTGGCCATAAGCAC
P2Y_4_	forward: GTGTGCACCGCTACATGGGCA
	reverse: TCAGGCAGAGTCGTGTCATGGCA
P2Y_6_	forward: AGGGGACCACTGGCCCTTCG
	reverse: TACCCAAGCAGCACGGCGAC
P2Y_12_	forward: AACTCTATCGGTCTTATGTCA
	reverse: AGAATACAGCAATGATGATGAA
P2Y_13_	forward: GGGCCTCATCGCTTTCGACAGG
	reverse: TCACGGATGATGGCGTTGCCT
P2Y_14_	forward: GGGGCGGAAGTGGCACAAGG
	reverse: GCGGCTGGACTTCCTCTTGACG
β-actin	forward: GTACGCCAACACAGTGCTG
	reverse: CGTCATACTCCTGCTTGCTG

### Degranulation

Bone marrow mast cells were incubated with IgE (Sigma-Aldrich, St. Louis, USA) to activate overnight, then washed by Tyrode's buffer [45]. The mast cells were resuspended in Tyrode's buffer at 2 × 10^5^ per well in a 96-well plate and stimulated with UDP, ovalbumin, or UDP + ovalbumin for 1 h. After that, the mast cells were treated with RIPA cell lysis buffer and supernatants were collected after centrifugation. These supernatants and cell culture medium supernatants were mixed with 0.1 M sodium citrate buffer (pH 4.5) including p-nitrophenyl N-acetyl-β-D-glucosamide (Sigma-Aldrich, St. Louis, USA) for 90 min at 37°C. The reaction was stopped by addition of 0.2 M glycine (pH 10.7). The absorbance of β-hexosaminidase released from mast cells was read at a wavelength of 405 nm.

### Chemotaxis assays

Chemotactic responses of mast cells were examined in 24-well Transwell plates (BD Falcon, USA) with 8-μm-pore-size polycarbonate filters. Cells (2 × 10^6^ cells/mL) in 200 μL RPMI 1640 were added to the top wells with or without UDP. The migration buffer containing PBS or 100 μm UDP was added to the lower wells. After 3 h at 37°C in 5% CO_2_, the mast cells in the lower chambers were harvested by centrifugation and resuspended with 100 μL PBS. The cells were counted on a blood cell counting plate.

### Statistical analysis

Significant differences were assessed with the *t*-test for comparisons between two groups and ANOVA test for comparisons among more than two groups. The data are presented as the mean ± SEM. All statistical values were measured using GraphPad Prism version 5.0 (GraphPad Prism Software, USA). Results were considered significant at *P* < 0.05.
